# The Effect of Glyphosate on Human Sperm Motility and Sperm DNA Fragmentation

**DOI:** 10.3390/ijerph15061117

**Published:** 2018-05-30

**Authors:** George Anifandis, Katerina Katsanaki, Georgia Lagodonti, Christina Messini, Mara Simopoulou, Konstantinos Dafopoulos, Alexandros Daponte

**Affiliations:** 1 Department of Obstetrics and Gynecology, ART Unit, School of Health Sciences, Faculty of Medicine, University of Thessaly, Larisa 41222, Greece; kakats1992@yahoo.gr (K.K.); georgia_lag@hotmail.com (G.L.); pireaschristina@gmail.com (C.M.); kdafop@med.uth.gr (K.D.); dapontea@otenet.gr (A.D.); 2 Department of Physiology, School of Health Sciences, Faculty of Medicine, Kapodistrian University of Athens, Athens 11225, Greece; marasimopoulou@hotmail.com

**Keywords:** sperm motility, sperm DNA fragmentation, glyphosate

## Abstract

Glyphosate is the active ingredient of Roundup^®^, which is one of the most popular herbicides worldwide. Although many studies have focused on the reproductive toxicity of glyphosate or glyphosate-based herbicides, the majority of them have concluded that the effect of the specific herbicide is negligible, while only a few studies indicate the male reproductive toxicity of glyphosate alone. The aim of the present study was to investigate the effect of 0.36 mg/L glyphosate on sperm motility and sperm DNA fragmentation (SDF). Thirty healthy men volunteered to undergo semen analysis for the purpose of the study. Sperm motility was calculated according to WHO 2010 guidelines at collection time (zero time) and 1 h post-treatment with glyphosate. Sperm DNA fragmentation was evaluated with Halosperm^®^ G2 kit for both the control and glyphosate-treated sperm samples. Sperm progressive motility of glyphosate-treated samples was significantly reduced after 1 h post-treatment in comparison to the respective controls, in contrast to the SDF of glyphosate-treated samples, which was comparable to the respective controls. Conclusively, under these in vitro conditions, at high concentrations that greatly exceed environmental exposures, glyphosate exerts toxic effects on sperm progressive motility but not on sperm DNA integrity, meaning that the toxic effect is limited only to motility, at least in the first hour.

## 1. Introduction

Glyphosate [*N*-(phosphonomethyl)-glycine] is the active ingredient of Roundup, acting as an inhibitor of the enzyme 5-enolpyruvylshikimate-3-phosphate synthase by interrupting the synthesis of essential aromatic amino acids in plants [1]. The extensive use of glyphosate as a risk for reproductive health has been and still is a controversial issue. Glyphosate, as well as aminomethylphosphonic acid (AMPA), the major breakdown product of glyphosate, are not developmentally toxic since it has been found that there were no adverse effects of glyphosate on mammalian reproduction [2]. Another study has described the negative impact of glyphosate on frog and chicken development [3]. A meta-analysis relative to the toxic effects of glyphosate revealed that exposure to glyphosate decreased sperm concentration in both mice and rats, indicating adverse effects on reproductive parameters [4]. Moreover, the harmful effect of glyphosate on sperm motility, mitochondrial functionality and sperm DNA integrity has been demonstrated in zebrafish [5]. Glyphosate at a dose of 5 mg/kg exerts deleterious effects on the sperm quality of Wistar rats [6], while recently it was reported that sperm parameters such as motility and concentration represent the most sensitive parameters affected by glyphosate in live-bearing *Jenynsia multidentata* [7]. Glyphosate in a dose-dependent manner was accompanied by an increase in abnormal and dead spermatozoa, implying that the effects on sperm quality may be due to the direct cytotoxic effect of glyphosate on spermatogenesis and/or indirectly via hypothalamic-pituitary-testis axis which controls reproductive efficiency [8]. The available literature regarding the potential exposure of humans to glyphosate provides evidence of extremely low exposures (with estimated doses >500-fold less than the recommended oral reference dose for glyphosate) and indicates that there is no solid evidence linking ambient exposure to glyphosate to adverse reproductive effects [9]. Nevertheless, various glyphosate formulations seem to have adverse effects on hormonal function, and it has been demonstrated that glyphosate-based herbicides, such as Roundup, have an impact on oxygen reactive species, and it also changes the redox system, resulting therefore in apoptosis [10,11]. Last, Roundup has been demonstrated to have deleterious effect on steroidogenesis and proliferation of bovine granulosa cells [12], indicating indirectly the impact of glyphosate on female gametes [13].

The present study is following the aim and scope of previous work concerning the effect of Roundup on human sperm motility and sperm mitochondria [14]. Considering the correlation between sperm motility and mitochondrial functionality [15], the aim of the present work is to elucidate and differentiate the role of Roundup from its main constituent, glyphosate. Additionally, we investigated the effect of glyphosate on SDF, a characteristic with a major role among the sperm parameters. 

## 2. Materials and Methods

### 2.1. Human Subjects

Thirty (30) healthy men volunteered for semen analysis for the study during 2016 and gave written informed consent; Institutional Review Board approval of the study was also obtained (protocol number: 2538/15.6.2016). The present study is following previous work concerning the effect of Roundup on human sperm motility and sperm mitochondria [14]. All of the subjects were living in the agricultural region of Greece, Thessaly. Sixty six percent of the study group (20/30) were employed in an office job, 16.7% (5/30) were farmers and 16.7% (5/30) were unemployed. A total of 16.7% (5/30) were married.

### 2.2. Sperm Collection and Preparation

Thirty fresh semen samples were collected after 48 h to 96 h of abstinence and were allowed to liquefy at 37 °C for 15–20 min. Semen analysis of each specimen was performed in terms of semen volume and sperm concentration determination in combination with the percentage of progressive motile (PRM), non-progressive motile (NPM) and immotile (IM) spermatozoa, according to WHO 2010 guidelines. Portions of 0.5 mL of the total volume of each specimen were centrifuged at 2000 rpm for 5 min and the supernatant from each sample was carefully discarded, while the pellet was resuspended in 1 mL of pre-warmed buffer solution (Gamete Buffer, William Cook Australia PTY LTD^®^, Brisbane, Australia) containing glyphosate at a final concentration of 0.36 mg/L. The rationale for the dose of 0.36 mg/L glyphosate was that in the previous work [14] we used 1 mg/L Roundup at final concentration which corresponded to 0.36 mg/L glyphosate. Therefore, the obtained results from glyphosate will be at least explanatory when compared to the results obtained by Roundup. Nevertheless, the proposed reference doses for glyphosate is 0.5 mg per kg body weight for acceptable daily intake (ADI)and 0.1 mg per kg body weight per day for the acceptable operator exposure level (AOEL). An aliquot of each initial fresh semen sample was kept aside for subsequent sperm analysis and served as control (C). After 1 h incubation, in the presence or absence of glyphosate, at room temperature, sperm analyses and measurement of sperm DNA fragmentation were performed in both control and glyphosate-treated semen samples. Both control and glyphosate-treated samples were subjected to the same experimental procedure. Moreover, the incubating conditions remained the same as in the previous work in order not to deviate from the experimental conditions used in the previous study. All samples for the purpose of the experiment were normalized to 10^6^ sperm cells in order to avoid any bias relative in the specific concentration of glyphosate to different sperm concentration.

### 2.3. Evaluation of DNA Fragmentation

Sperm DNA fragmentation was evaluated with the use of the Halosperm^®^ G2 Kit. It is a Sperm Chromatin Dispersion (SCD) method which is mainly based on the presence or the absence of the halo appearance after appropriate processing. Spermatozoa that demonstrate big and medium size halos were considered as without DNA fragmentation, while spermatozoa with small and no halos were considered as fragmented. Degenerative spermatozoa may also exist, and were counted as fragmented. The sperm DNA fragmentation index (SDFI) in each sperm sample was calculated by the formula: SDF (%) = Fragmented + Degenerated/Total cells counted × 100. For the purpose of the study, a minimum of 200 spermatozoa per sample were scored under the ×100 objective of the microscope. To reduce any bias, two different experts counted at least 150 spermatozoa each. The whole process has been described in detail elsewhere [16,17]. Briefly, an aliquot of the semen sample was diluted to 20 × 10^6^/mL in phosphate-buffered saline (PBS). Eppendorf tubes were placed in a water bath at 90–100 °C for 5 min in order for the agarose to melt. After 5 min of incubation, we transferred 50 μL of the diluted semen sample to the 100-μL melted agarose tube and we mixed gently with a pipette. We placed 8 μL of the cell suspension onto the sample well and it was covered with a coverslip. Slides were then placed on a gold plate in the refrigerator (4 °C) for 5 min so that the agarose solidified with the sperm cells embedded within. The coverslips were gently removed and the slides were placed horizontally in an elevated position. We then applied the denaturant agent, making sure the slide was fully covered, and we incubated it for 7 min. Then, the slides were horizontally immersed in a lysis solution and incubated for 20 min. After washing with abundant distilled water for 5 min, the slides were dehydrated in increasing concentrations of ethanol (70% and 100% for 2 min each) and then were air-dried. Slides were washed and allowed to dry. Strong staining is preferred to visualise the periphery of the halos of the dispersed DNA loops.

Demographic data, sperm characteristics (volume, concentration, motility and DNA fragmentation) were normally distributed (one sample Kolmogorov–Smirnov test) and statistical analysis was performed with the paired *t*-test and with the non-parametric Wilcoxon–Mann–Whitney test whenever it was appropriate. The level of 0.05 was used to determine statistical significance. Numeric values were expressed as mean ± SEM. The statistical software package SPSS v.17 was used.

## 3. Results

**Figure 1:**
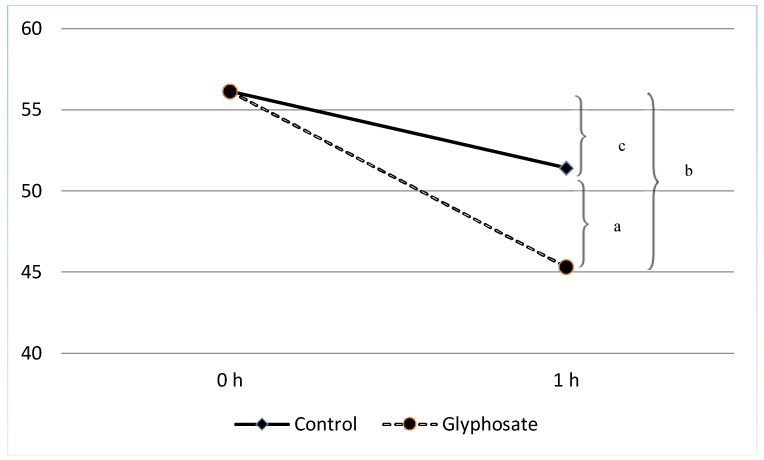
Percentage of progressive motility (PRM) between glyphosate-treated (*n* = 30) and control sperm samples (*n* = 30). Progressive motility of glyphosate-treated sperm samples was significantly lower compared to control samples (51.4% ± 2.4% vs. 45.3% ± 2.6%, a < 0.05) 1 h post-treatment. The reduction (5.3%) between zero time and 1 h post-treatment in the control samples was not significant (c > 0.05), while the reduction (11.4%) between zero time and 1 h post-treatment in glyphosate-treated samples was significant (b < 0.05).

Demographic data and basic sperm parameters at zero time of all the men studied are shown in Table 1. Table 2 shows the sperm characteristics in terms of motility and the various percentages of DNA fragmentation (medium, small, no halos and degenerative) between un-treated and glyphosate-treated spermatozoa 1 h post-treatment. The reduction in progressive motility of control samples (5.33%) was not significant (c > 0.05) in contrast to the reduction in glyphosate-treated sperm samples (11.43%) which reached significant levels (b < 0.05) between zero time and 1 h post-treatment (Figure 1). The progressive motility of glyphosate-treated sperm samples was significantly lower compared to control samples (51.43% ± 2.4 vs. 45.33% ± 2.6, a < 0.05) at 1 h post-treatment. In comparison, in our previous work the progressive motility reduction in Roundup-treated sperm samples 1 h post-treatment was 18.1% (from 53.5 to 35.4), while at the same time period the reduction in un-treated controls was 7.1% (from 53.5 to 46.4) [14].

## 4. Discussion

In the present study, the in vitro effect of glyphosate on human sperm motility and SDF is demonstrated for the first time. Motility is one of the main aspects in respect to fertilization, while SDF have been found to play a major role in normal fertilization and subsequent pre- and post-implantation development. Although an assessment of various doses of glyphosate would determine the exposure level more accurately, in the present study after 1 h exposure of spermatozoa to 0.36 mg/L glyphosate, a significant decrease in sperm progressive motility was observed, which was not accompanied by an increase in SDF compared to control sperm cells.

A screening analysis of various pesticides in semen samples is needed in order to characterize the environmental exposure of each sample at the beginning of the experiment and to rule out a possible effect of a previous exposure to pesticides. Moreover, a problem with most toxicological studies is obtaining a clear relationship between baseline cytotoxicity and in vivo toxicity, hence, the physico-chemical properties of the compound or the biotransformation process cannot be taken into account. Therefore, the extrapolation of a toxic concentration in the in vitro system to a toxic dose in the in vivo situation is difficult or impossible in most cases. Nevertheless, in the present study a significant reduction in progressive motility of the glyphosate-treated sperm samples was observed. The observed reduction (11.4%) was very similar to the reduction demonstrated in a the previous work with Roundup-treated sperm samples (18.1%) [14]. As was proposed in the previous work, the present reduction may be due to mitochondrial functionality. If glyphosate and Roundup exhibit very similar if not identical effects on sperm motility we may assume that the effect of Roundup is coming mainly from glyphosate, although the presence of surfactants in Roundup means that their toxic effects cannot be ruled out [18]. In contrast to the German Federal Institute for Occupational Safety and Health [19], which provided evidence that there is no adverse reproductive effect on rats exposed to high doses of glyphosate and the European Food Safety Authority [20] that showed that glyphosate is unlikely to be toxic for reproduction or development, the present study demonstrates that glyphosate in the concentration used has significant impact on male gametes, in terms of motility. The possible explanation for this difference is based mainly on the fact that in the present study, glyphosate administration was rather acute since it was an in vitro experiment. In a recent publication where the authors measured the urinary excretion levels of both glyphosate and its metabolite aminomethylphosphonic acid (AMPA), it was reported that mean glyphosate urine levels increased over time and reached 0.45 μg/L during the 2014–2016 period [21]. Moreover, in a review article concerning the glyphosate findings in human urine samples, it was proposed that glyphosate exposure was far lower than the acceptable daily intake (ADI) or the acceptable operator exposure level (AOEL) [22]. In other studies, glyphosate effects through air consumption, where biochemical agents or enzymes are mediated were evaluated. Thus, glyphosate might be catalyzed before it reaches male gametes, making the effect of glyphosate less toxic. Specifically, in a study conducted in Colombia for environmental and human health assessment, it was observed that aerially applied glyphosate did not exert any significant risk on both human health and the environment [23,24]. Nevertheless, in a very recent study, a total loss of sperm motility was observed in yellowtail tetra fish *Astyanax lacustris*, after exposure to low concentrations of glyphosate-based herbicide [25], although it should be mentioned that there are also other parameters such as testicular tissue volume or organ blood flow rates that are quite different in the tetra fish *Astyanax lacustris*.

Bearing in mind that it is possible that small molecule toxicant products may reach concentrations in semen similar to those in blood or urine plasma [26], glyphosate itself or its metabolite AMPA may induce adverse effects on various sperm parameters, including motility and sperm DNA fragmentation. So far, data concerning the effect of toxic agents on human sperm DNA integrity are scarce. SDF is a basic parameter of sperm analysis, which has been routinely applied in many ART Centers [27,28]. The positive correlation between SDF and miscarriage rate is well known [29]. Spermatozoa with increased DNA fragmentation might not provide intact sperm DNA to the developing embryo after fertilization, which may cause early pregnancy loss or even degeneration of the embryo before reaching the implantation point. Therefore, due to the significant contribution of paternal genetic material to embryo development, DNA fragmentation should be taken seriously as a main parameter of sperm function. Nevertheless, SDF tests should be offered to infertile men with evidence of exposure to pollutants or toxicants [28]. In the present work we investigated the possible effect of 0.36 mg/mL glyphosate on SDF after 1 h of treatment. It was observed that in the first hour, glyphosate did not exert any significant effect on SDF relative to untreated controls, something which is in line with a study which demonstrated that glyphosate alone has low toxicity on the male reproductive system of rats [30]. An explanation for this observation is that nuclear de-condensation follows motility impairment, which seems to be the first strike on spermatozoa. Immediately after, a large part of the sperm sample loses its ability to move and a few hours later all spermatozoa lose their DNA integrity. In the present study, the impact of glyphosate on DNA integrity was evaluated 1 h post-treatment with glyphosate. Given the fact that reduced motility was not linked with similar loss of DNA integrity, it seems that glyphosate at the concentration used exerts toxic effect on sperm motility but not on sperm DNA integrity, meaning that the toxic effect is limited only to motility, at least in the first hour. Nevertheless, a toxic effect after 2 or 3 h post-treatment cannot be excluded. Moreover, sperm DNA fragmentation is related to sperm staining patterns and the methods applied [31,32]. A small preliminary study of five samples conducted by our team, comparing the percent of DNA fragmentation between glyphosate- and Roundup-treated spermatozoa, found that the impact of both substances on SDF was comparable (data not shown). Although solid conclusions cannot be drawn from this preliminary study, it can be assumed that both substances demonstrate similar effects.

In conclusion, the present study is the first study that investigates the impact of glyphosate on sperm motility and SDF. Glyphosate at the concentration used, exerted negative effects on sperm motility but there was no similar effect on DNA integrity. Moreover, given the comparable effects of glyphosate and Roundup on motility and SDF it can be assumed that Roundup exerts its action through glyphosate, which is the main constituent of Roundup.

## Figures and Tables

**Table 1:** Demographic data of all men studied and basic sperm parameters at zero (0) time.

Variable	Value (Mean ± SEM)
No of samples	30
Age (years)	40.9 ± 1.5
BMI (Kg/m^2^)	28.3 ± 0.9
Abstinence (days)	3.3 ± 0.1
Volume (mL)	3.3 ± 0.2
Concentration (mil/mL)	56.7 ± 7.9
PRM (%)	56.1 ± 3.1
NPM (%)	11.1 ± 1.1
IM (%)	32.7 ± 3.1

BMI: Body Mass Index; PRM: progressive motility; NPM: non-progressive motility; IM: immotility.

**Table 2:** Mean ± SEM values of sperm parameters studied in both control and glyphosate-treated samples after 1 h of treatment.

Variables	Values (Mean ± SEM)		*p* Value
	Control (1 h)	Glyphosate (1 h)	
No of samples	30	30	
PRM (%)	51.4 ± 2.4	45.3 ± 3.6	<0.05
NPM (%)	9.4 ± 1.1	8.5 ± 1.1	NS
IM (%)	39.2 ± 2.6	46.2 ± 3.7	<0.05
Small halos (%)	8.2 ± 1.3	8.0 ± 0.8	NS
No halos (%)	25.4 ± 2.7	28.8 ± 3.1	NS
Degenerative (%)	15.2 ± 2.8	14.3 ± 1.4	NS
SDF (%)	48.8 ± 4.2	51.1 ± 4.1	NS

PRM: progressive motility; NPM: non-progressive motility; IM: immotility; SDF: sperm DNA fragmentation; NS: not significant.
